# Refining a Digital Therapeutic Platform for Home Care Agencies in Dementia Care to Elicit Stakeholder Feedback: Focus Group Study With Stakeholders

**DOI:** 10.2196/32516

**Published:** 2022-03-02

**Authors:** Aaron Gilson, Michele Gassman, Debby Dodds, Robin Lombardo, James H Ford II, Michael Potteiger

**Affiliations:** 1 Social & Administrative Pharmacy Division School of Pharmacy University of Wisconsin–Madison Madison, WI United States; 2 Generation Connect York, PA United States

**Keywords:** dementia, technology, mobile app, home care, focus groups, qualitative research, digital therapeutics, value-based care, aging in place, caregiving

## Abstract

**Background:**

Persons living with dementia require increasing levels of care, and the care model has evolved. The Centers for Medicare and Medicaid Services is transitioning long-term care services from institutional care to home- or community-based services, including reimbursement for nonclinical services. Although home care companies are positioned to handle this transition, they need innovative solutions to address the special challenges posed by caring for persons living with dementia. To live at home longer, these persons require support from formal caregivers (FCGs; ie, paid professionals), who often lack knowledge of their personal histories and have high turnover, or informal caregivers (eg, family or friends), who may have difficulty coping with behavioral and psychological symptoms of dementia. The Generation Connect platform was developed to support these individuals and their formal and informal caregivers. In preliminary studies, the platform improved mood and influenced caregiver satisfaction. To enhance platform effectiveness, Generation Connect received a grant from the National Institutes of Health Small Business Innovation Research to improve clinical outcomes, reduce health care costs, and lower out-of-pocket costs for persons living with dementia who receive care through home care agencies.

**Objective:**

This study aims to evaluate information elicited from a series of stakeholder focus groups to understand existing processes, needs, barriers, and goals for the use of the Generation Connect platform by home care agencies and formal and informal caregivers.

**Methods:**

A series of focus groups were conducted with home care agency corporate leadership, home care agency franchise owners, home care agency FCGs, and informal caregivers of persons living with dementia. The qualitative approach allowed for unrestricted idea generation that best informed the platform development to enable home care providers to differentiate their dementia care services, involve informal caregivers, improve FCG well-being, and extend the ability of persons living with dementia to age in place. Using the Technology-Enabled Caregiving in the Home framework, an inductive and iterative content analysis was conducted to identify thematic categories from the transcripts.

**Results:**

Overall, 39 participants participated across the 6 stakeholder focus groups. The following five overarching themes were identified: technology related; care services; data, documentation, and outcomes; cost, finance, and resources; and resources for caregivers. Within each theme, the most frequent subthemes were identified. Exemplar stakeholder group statements provided support for each of the identified themes.

**Conclusions:**

The focus group results will inform the further development of the Generation Connect platform to reduce the burden of caregiving for persons living with dementia, evaluate changes in cognition, preserve functional independence, and promote caregiver engagement between these individuals. The next step is to evaluate the effectiveness of the revised platform in the National Institutes of Health Small Business Innovation Research phase 2 clinical trial to assess the efficacy of its evidence-based interventions and market viability.

## Introduction

### Background

Owing to the degenerative nature of the disease, persons living with dementia require escalating support for their care and are increasingly vulnerable to institutionalization. The historical model for providing care to persons living with dementia has involved the heavy use of facility-based care at great financial and social costs to the Centers for Medicare and Medicaid Services and persons living with dementia and their families [1,2]. On average, the total per-person Medicaid payments for persons living with dementia aged >65 years are 23 times higher than Medicaid payments for other Medicare beneficiaries [1]. In 2020, the national cost of caring for people with Alzheimer disease and related dementias (ADRD) is projected to reach US $305 billion, with 67% (US $206 billion) paid by Medicare and Medicaid to cover health care and long-term care payments for people with ADRD [1]. As such, over 50% of persons living with dementia die in nursing homes or medical facilities [3].

### Caregiver Roles for Persons Living With Dementia

Persons living with dementia overwhelmingly want to age in place and avoid institutionalization [4]. To live at home longer, persons living with dementia require support from (1) formal or clinical caregivers (ie, paid professionals), who often lack knowledge of persons living with dementias’ personal histories and have high turnover rates, or (2) informal or nonclinical caregivers (eg, family or friends), who may have difficulty coping with behavioral and psychological symptoms of dementia (eg, aggression and anxiety) [5]. To address this need, the Centers for Medicare and Medicaid Services is transitioning long-term care services from institutional care to home- or community-based services, including expanding Medicare Advantage (MA) plans to include nonclinical services as reimbursable supplemental benefits [6].

The importance of those who engage with persons living with dementia to deliver such care services will become amplified as the demand for nonclinical home care services increases worldwide. Such expansion of services is certainly welcome, as evidence currently supports the benefits of nonclinical home care providers on patient outcomes [7]. Research findings demonstrate that nonclinical providers can reduce behavioral and psychological symptoms of dementia and health care costs through consistency of care and person-centered engagement [8]. However, home care providers reported a mean caregiver turnover rate of 82% in 2018 [9], which often translates into prevalent service disruptions. In addition, home care providers lack solutions to ensure that frontline nonclinical caregivers have the necessary knowledge of persons living with dementia’s personal histories to implement evidence-based care methods [9]. Even with appropriate knowledge, nonclinical providers typically lack systems and processes to demonstrate improved clinical outcomes and cost savings [10,11]. This current struggle represents a promising opportunity for nonclinical home care providers to have access to user-friendly, easily implementable data collection tools.

The use of information communication technologies (ICTs) by frontline care staff to enhance patient care and record clinical outcomes is common in hospitals, long-term care facilities, and skilled home health care [12]. However, ICT use by frontline caregivers in nonclinical home care is rare. A small number of providers are exploring solutions for family connectivity and remote care; however, there have been no known technology solutions for frontline caregivers that specifically address the use of digital therapeutics to enhance social engagement and data collection for both persons living with dementia and their caregivers.

### Development of a Technology-Based Data Collection Tool

**Figure 1:**
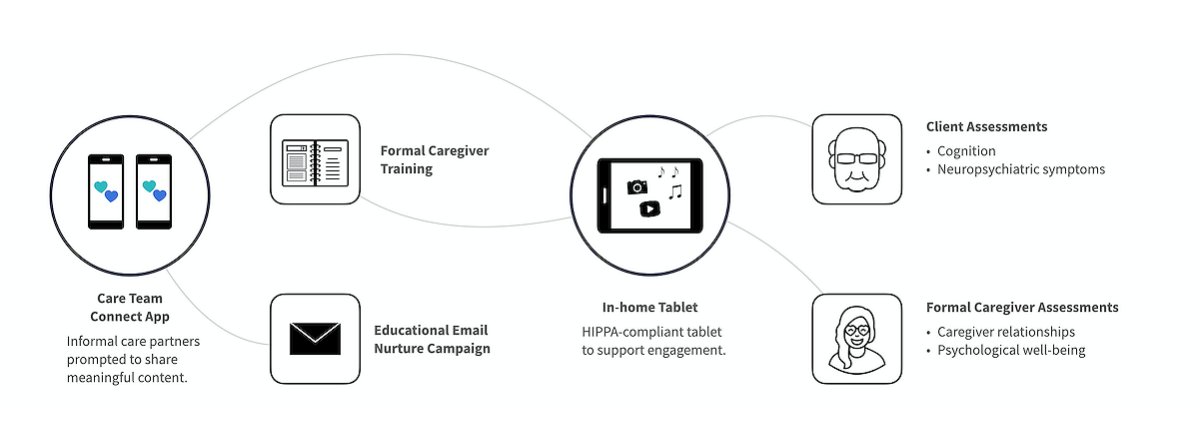
Generation Connect platform configuration. HIPPA: Health Insurance Portability and Accountability Act.

Generation Connect, a gerontology-focused software development company, developed a digital therapeutic platform to support informal and formal caregivers (FCGs) in the care of persons living with dementia (Figure 1).

The Generation Connect platform was originally conceptualized to enhance the informal caregiver’s understanding of evidence-based nonpharmacological interventions and support FCGs in facilitating person-centered care. The Generation Connect platform focuses on the following three key technology initiatives to address barriers to aging in place: (1) the deployment of specially configured tablets to enhance persons living with dementia’s engagement routines, (2) the development of an application to improve collaboration between home care providers and informal caregivers, and (3) the creation of assessment tools to streamline data collection related clinical outcomes for persons living with dementia and their caregivers. The Generation Connect platform is intended for its design to be used across all stages of dementia. Current users tend to be in the moderate to later stages of dementia; however, future efforts are being planned to broaden its usage for persons living with dementia in the earlier stages.

Generation Connect partnered with the University of Wisconsin–Madison to determine how care providers’ use of computer tablets to engage persons living with dementia improved their mood and influenced caregiver satisfaction [13,14]. This preliminary research motivated further exploration of (1) complications saving client preferences, (2) the inability to digitally engage informal caregivers while maintaining the Health Insurance Portability and Accountability Act (HIPAA) compliance, and (3) the lack of systems for collecting clinical data.

To begin addressing the insights gained from these preliminary studies, Generation Connect received a feasibility grant funded by the National Institute on Aging from the National Institutes of Health Small Business Innovation Research (NIH SBIR) phase 1. The purpose of this grant was to develop an innovative technology solution that could eventually help improve clinical outcomes, reduce health care costs, and lower out-of-pocket costs for persons living with dementia who receive care through home care agencies. Through the NIH SBIR grant, Generation Connect sought to enhance the platform to address the following three key technology initiatives: (1) develop platform features that prompt care teams (informal caregivers and FCGs) to participate in evidence-based engagement strategies (eg, music, reminiscing, and socialization), (2) deploy HIPAA-compliant tablets to help care teams personalize these engagement routines, and (3) integrate clinically validated assessment tools into care routines.

### Objectives

As part of the initial exploration and discovery phase of this grant, a series of focus groups were conducted with key project stakeholders, including (1) home care agency corporate leadership, (2) home care agency franchise owners, (3) home care agency FCGs, and (4) informal caregivers of persons living with dementia. The purpose of this phase of the project is to evaluate the information elicited from the series of stakeholder focus groups conducted by the Generation Connect design team to develop a better understanding of existing processes, needs, barriers, and goals for the use of a digital therapeutic platform by home care agencies and informal caregivers.

## Methods

### Overview

A formal qualitative approach [15] allowed for the unrestricted generation of ideas that would best position the Generation Connect platform to enable home care providers to differentiate their dementia care services, involve informal caregivers, improve FCG well-being, and extend the ability of persons living with dementia to age in place. Focus group participants completed an electronic survey before engaging in the focus group sessions, which included a section explaining the study and requiring their consent to participate.

### Ethics Approval

This study was approved by the WCG institutional review board (IPCDCDA-2020).

### Focus Group Participants

The following stakeholder categories represented the focus group participants:

- Franchise owners (organization A): it was a national home care agency with 565 independently owned and operated units. A partnership with Generation Connect in 2018 involved an alpha pilot to deploy the Generation Connect platform in 10 markets, which continues to be used in clients’ homes. These owners also participated in testing the platform and recruited clients for this NIH SBIR project. These home care leaders had direct experience in implementing the Generation Connect platform as part of their home care services. These individuals provided unique insights into how the Generation Connect platform influenced clinical outcomes for clients and FCG, business outcomes, and FCG satisfaction and company culture.
- FCGs (organization A): it was a group of 15 professional caregivers with experience using the Generation Connect platform as part of the alpha pilot mentioned in item 1. These frontline care workers had direct experience using the Generation Connect platform among people with dementia. FCG often lacked a personal relationship with the people with dementia but had more dementia-related experience than other FCGs.
- Informal caregivers (receiving services from organization A): it was a group of 4 women caring for their fathers with memory loss who have experience using the Generation Connect platform as part of their loved one’s care. These stakeholders could be seen as more directly reflecting the perspective of the person with dementia, as recruiting people with dementia was unfeasible for this study. Family caregivers have extensive, and often complex, relationships with the care recipient and other family members. These family members had direct experience using the Generation Connect platform as part of receiving home care services and provided a valuable perspective on how Generation Connect platform use could affect home care services.
- Franchise owners (organization B): it was a medical and nonmedical home care provider, with over 500 independently owned and operated units in the United States and 130 abroad. These home care leaders did not have experience using the Generation Connect platform but had agreed to participate in phase 1 pilot testing. They were specially selected by the corporate leadership team because of their past experience in implementing caregiver technologies and specialty dementia care programs. They can provide unique insights regarding the challenges of clinical implementation, business strategy, and impact on company culture for such programs. These owners also participated in testing the Generation Connect platform and recruited clients for the NIH SBIR project.
- Corporate leadership team (organization B): Generation Connect partnered with organization B to recruit owners for participation in phase 1 activities to deploy and pilot test the Generation Connect platform. Similar to organization A, they are one of the largest franchisors (≥650 locations) and have experience piloting caregiver technology initiatives across their franchise network.
- Corporate leadership team (organization C): a national home care franchisee, with ≥600 independently owned and operated units in the United States and nearly 480 abroad. Generation Connect had been involved with organization C on consulting projects involving senior technology, but the organization had no previous experience using the Generation Connect platform. As one of the largest home care franchisors (≥1100 locations), with extensive experience in implementing national tech initiatives, they can provide unique insights into moderators and mediators of widescale Generation Connect platform adoption across the franchise network.

### Participant Recruitment

Participants with direct knowledge of existing processes, needs, and barriers related to dementia care in the home were purposefully selected to participate in the focus group sessions. For the corporate focus groups, Generation Connect sent an invitation to our primary point of contact from organizations B and C, inviting corporate leaders to participate. Both organizations are national leaders in nonmedical home care and have recent experience implementing technology initiatives at the corporate level. Our contacts recruited colleagues that provided the most helpful insights. It was an open invitation for corporate leadership within the organization. Corporate leaders from organization B helped recruit franchise owners from within the network to participate in focus group and product testing. For the franchise owners’ focus groups, we invited all home care owners who opted to participate in phase 1 pilot testing. It was an open invitation to local franchisee leadership staff. Each organization had at least one director-level employee who participated in the focus groups. For the FCG focus group, home care leadership invited 17 FCGs with experience using the Generation Connect platform to be involved in the focus group; of the 17 FCGs, 15 (88%) decided to participate. For the informal caregiver group, each of the 4 organization A owners involved in phase 1 offered 1 family member with previous experience using the platform the opportunity to participate in the focus group, and all 4 accepted.

All participants from organization A, including franchise owners, FCGs, and informal caregivers, had firsthand experience using a pilot version of the Generation Connect platform. Although the franchise owners of organization B had no previous experience using the Generation Connect platform, they had experience implementing a variety of dementia care and technology initiatives.

### Interview Guide Development

**Figure 2:**
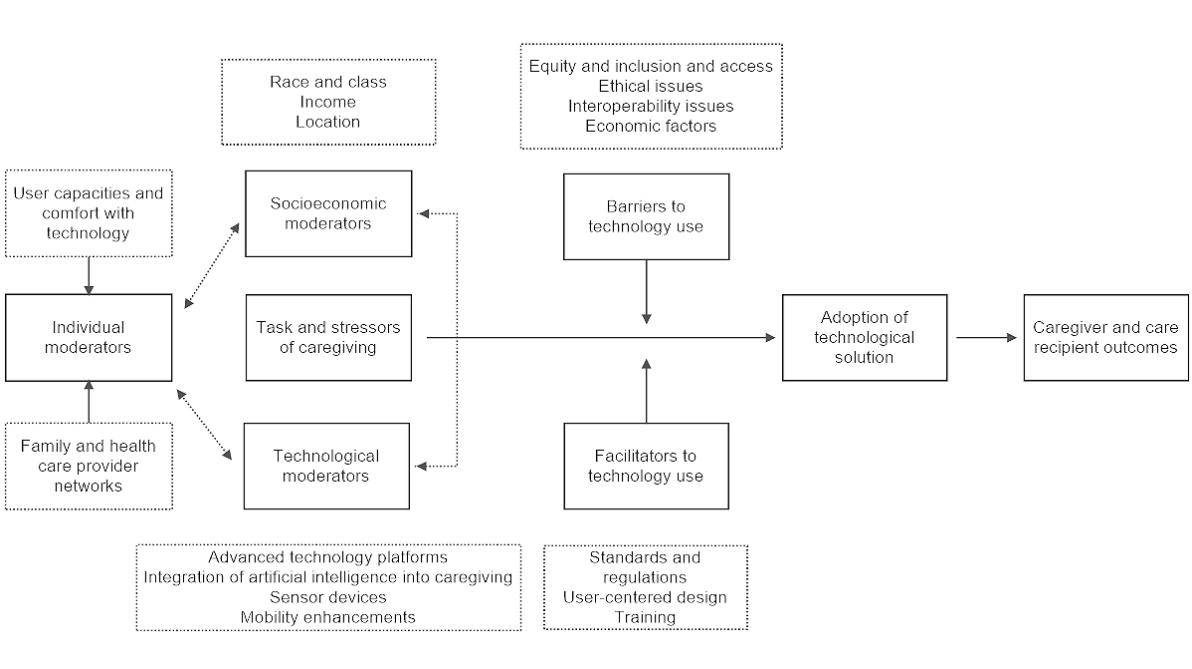
Technology-Enabled Caregiving in the Home framework (adopted from a study by Lindeman et al [16]).

Interview guides for each stakeholder group were developed by the research team. Guide development was informed by Technology-Enabled Caregiving in the Home (TECH) [16], which is the theoretical framework that guides this research (Figure 2).

The TECH framework examines the characteristics that could influence FCGs’ adoption of technological platforms that, in turn, can contribute to persons living with dementia and caregiver outcomes. Thematic model domains include (1) individual, socioeconomic, or technical moderators; (2) barrier or facilitator mediators; and (3) technology-related measurement issues. This model provides a *map of thinking* as the basis for broader adoption of caregiving solutions [16]. Influencing caregiving through such moderating and mediating pathways to improve caregiving outcomes has the benefit of promoting technological solutions throughout the system of caregiving.

The interview guides were distributed internally for review, involving team members without clinical or domain knowledge, to ensure that they were easy to comprehend and interpreted uniformly. Then they were refined based on reviewers’ feedback. This refinement process involved nonsubstantive improvements in readability and understandability to prompt unequivocal responses more directly from participants. The resulting guides were structured for 1-hour–long interviews.

### Focus Groups

Between November 2020 and January 2021, the Generation Connect staff conducted a total of 6 focus groups with stakeholders. For confidentiality and proprietary reasons, focus groups with home care corporate leaders and home care franchise owners and case managers were conducted separately. This separation helped ensure that members of both stakeholder groups would feel more comfortable in freely sharing information about their company. Owing to the COVID-19 pandemic, all focus groups were conducted via a HIPAA-compliant version of GoTo's GoToMeeting, an internet-based meeting platform. Dates and times for interviews (conducted by MP or DD) were scheduled based on the convenience of each participant. The interviews of focus group participants generally followed the structure of the focus group guides and questions related to the themes relevant to each stakeholder group (Table 1).

**Table 1:** Focus group question categories by recipient.

Focus group question categories	Question category included in the recipient focus group			
	Corporate leaders	Franchise owners and case managers	Formal caregivers	Informal caregivers
Person-centered dementia care or care service	✓	✓	✓	✓
Social determinants of health	✓			
Approach to Medicare Advantage	✓	✓		
Caregiver staff		✓		
Tracking outcomes		✓		
Living with dementia				✓
The role of technology in client care	✓	✓		
Technology-assisted caregiving			✓	✓

### Analysis of Interview Data

Focus group meetings were automatically transcribed by GoToMeeting. The transcript was reviewed, verified for accuracy and anonymity, and deidentified by the Generation Connect staff. All deidentified transcripts were then made available to the University of Wisconsin–Madison researchers through upload to a secure university network server to further control for data security and confidentiality. A general inductive and iterative content analysis approach was used to generate the development and identification of thematic categories from the interview data. These themes were derived according to the relevant TECH factors (individual, socioeconomic, or technical moderators; barrier or facilitator mediators; and technology-related measurement issues) and the various TECH variable domains within each factor. The inductive approach is a systematic procedure for analyzing qualitative data that are commonly used in health and social science research and evaluation [17]. An experienced qualitative coder (AG) independently coded all interview transcripts while developing a preliminary coding scheme to capture themes related to caregiver, patient and family, work system, and programmatic factors. Effort was made to ensure that the initial list of codes avoided overlap and reduced redundancy whenever practicable.

Once the transcripts were reviewed and coded, and all the codes and subcodes were compiled, they were transferred to another researcher (MG). The researcher then independently read the transcript to validate both the coding (ie, the interview content that was assigned to a code) and the list of codes. After the initial validation process, the researchers met to compare their coding results. The researchers sought consensus to resolve discrepancies in coder agreement to ensure more accurate coding; 100% consensus was achieved across all codes and subcodes. In the final stage of this coding process, the same 2 researchers again evaluated the extracted content within each code for consistency and were then forwarded to Generation Connect staff (MP) for review, which resulted in no additional changes and represented the final validation of the codes and subcodes and their application to the interview text. The final coding dictionary consisted of five main codes, with 40 subcodes across the main themes: care services, cost, finance, resources, data, documentation, outcomes, resources for caregivers, and technology.

This phase 1 study did not meet the criteria for an Applicable Clinical Trial; therefore, it was not registered on ClinicalTrials.gov.

## Results

### Overview

A total of 39 participants were interviewed during the 6 stakeholder focus groups. The following five overarching coding themes were identified and are presented here in order of frequency: (1) *technology related*; (2) *care services*; (3) *data, documentation, and outcomes*; (4) *cost, finance, and resources*; and (5) *resources for caregivers*. For each coding theme, the most frequent subthemes served as the key finding for this paper. In addition, after the broad themes and key findings are provided, a separate section titled *Other Relevant Findings* is offered to highlight content that was frequently elicited from a particular stakeholder group or that represented unique or noteworthy implications. Table 2 demonstrates the frequency with which the various themes and subthemes were represented and the extent to which individual stakeholder groups contributed to each.

Exemplar statements obtained through focus group transcripts are listed for each stakeholder group that offered feedback for a particular subtheme, to provide qualitative support for each of the coding domains. When necessary, interview participants’ quotations were edited to remove nonsubstantive interjections or repetitive words or phrases or connect partial phrases that were eventually stated in their entirety.

**Table 2:** All identified focus group themes and frequencies.

Identified theme			Corporate (n=196), n (%)		Formal caregiver (n=94), n (%)		Franchise owner (n=222), n (%)		Informal caregivers (n=38), n (%)		Total (n=550), n (%)
**Care services**			53 (27)		22 (23.4)		47 (21.2)		12 (31.6)		134 (24.4)
	Ability to react to changes	2 (3.8)		2 (9.1)		8 (17)		3 (25)		15 (11.2)	
	Addressing family needs	1 (1.9)		0 (0)		2 (4.3)		0 (0)		3 (2.2)	
	Assess and support cognition or mental health	12 (22.6)		0 (0)		6 (12.8)		0 (0)		18 (13.4)	
	Expanding care services	6 (11.3)		0 (0)		2 (4.3)		0 (0)		8 (6)	
	Individualized care	7 (13.2)		1 (4.5)		12 (25.5)		0 (0)		20 (14.9)	
	Maintaining patient in home	1 (1.9)		0 (0)		0 (0)		0 (0)		1 (0.7)	
	Patient and family engagement	15 (28.3)		2 (9.1)		12 (25.5)		1 (8.3)		30 (22.4)	
	Preparation for personal care rather than clinical care	4 (7.5)		0 (0)		1 (2.1)		0 (0)		5 (3.7)	
	Response to patient	1 (1.9)		17 (77.3)		4 (8.5)		8 (66.7)		30 (22.4)	
	Variability of care services	4 (7.5)		0 (0)		0 (0)		0 (0)		4 (3)	
**Cost, finance, and resources**			33 (16.8)		3 (3.2)		44 (19.8)		2 (5.3)		82 (14.9)
	Administrative burden	3 (9.1)		0 (0)		3 (6.8)		0 (0)		6 (7.3)	
	Cost issues	5 (15.2)		0 (0)		10 (22.7)		1 (50)		16 (19.5)	
	Cost options	11 (33.3)		1 (33.3)		1 (2.3)		0 (0)		13 (15.9)	
	Dedicated staff	2 (6.1)		0 (0)		5 (11.4)		0 (0)		7 (8.5)	
	Medicare Advantage or other programs	10 (30.3)		0 (0)		14 (31.8)		0 (0)		24 (29.3)	
	Need for resources	1 (3)		2 (66.7)		1 (2.3)		1 (50)		5 (6.1)	
	Turnover issues	1 (3)		0 (0)		10 (22.7)		0 (0)		11 (13.4)	
**Data, documentation, and outcomes**			38 (19.4)		8 (8.5)		36 (16.2)		7 (18.4)		89 (16.2)
	Demonstration of value	15 (39.5)		0 (0)		3 (8.3)		0 (0)		18 (20.2)	
	Demonstration of value (patience)	0 (0)		0 (0)		4 (11.1)		0 (0)		4 (4.5)	
	Documenting patient routines and incidences	1 (2.6)		2 (25)		1 (2.8)		7 (100)		11 (12.4)	
	Need to document outcomes	17 (44.7)		0 (0)		13 (36.1)		0 (0)		30 (33.7)	
	Need to document outcomes (standardization)	1 (2.6)		0 (0)		0 (0)		0 (0)		1 (1.1)	
	Need to document outcomes, with some limitations	1 (2.6)		0 (0)		0 (0)		0 (0)		1 (1.1)	
	Sharing information and resources	3 (7.9)		6 (75)		15 (41.7)		0 (0)		24 (27)	
**Resources for caregivers**			15 (7.7)		21 (22.3)		31 (14.0)		3 (7.9)		70 (12.7)
	Allocating caregiver services	2 (13.3)		0 (0)		7 (22.6)		0 (0)		9 (12.9)	
	Benefit of experience	0 (0)		6 (28.6)		5 (16.1)		0 (0)		11 (15.7)	
	Caregiver support and mentoring	0 (0)		0 (0)		5 (16.1)		0 (0)		5 (7.1)	
	Need for training and education	13 (86.7)		15 (71.4)		14 (45.2)		3 (100)		45 (64.3)	
**Technology**			57 (29.1)		40 (42.6)		64 (28.8)		14 (36.8)		175 (31.8)
	Acceptance of technology	6 (10.5)		3 (7.5)		7 (10.9)		1 (7.1)		17 (9.7)	
	Acceptance of technology and barriers to use	4 (7)		2 (5)		1 (1.6)		3 (21.4)		10 (5.7)	
	Acceptance of technology, but not universal	0 (0)		0 (0)		1 (1.6)		0 (0)		1 (0.6)	
	Benefit of family contributing content	4 (7)		1 (2.5)		3 (4.7)		3 (21.4)		11 (6.3)	
	Benefit of personalized content	7 (12.3)		11 (27.5)		11 (17.2)		3 (21.4)		32 (18.3)	
	Benefit of technology	19 (33.3)		10 (25)		20 (31.3)		1 (7.1)		50 (28.6)	
	Benefits of technology, mostly but not always	0 (0)		1 (2.5)		0 (0)		0 (0)		1 (0.6)	
	Benefits of technology, with some limitations	1 (1.8)		0 (0)		5 (7.8)		0 (0)		6 (3.4)	
	Challenges of technology	5 (8.8)		9 (22.5)		9 (14.1)		1 (7.1)		24 (13.7)	
	Expanding personalization options	2 (3.5)		3 (7.5)		1 (1.6)		2 (14.3)		8 (4.6)	
	Expanding technology	3 (3.5)		0 (0)		5 (7.8)		0 (0)		8 (4.6)	
	Integrating technology into standard care	6 (10.5)		0 (0)		1 (1.6)		0 (0)		7 (4)	

### Theme 1: Technology Related

#### Overview

The purpose of the focus group interviews was to elicit feedback that would ultimately help guide the development and refinement of a tablet-based innovative technology (the Generation Connect platform) to be used with patients with dementia. As a result, it is not surprising that technology-related content was the prevailing thematic area (occurring 175 times). The stakeholder focus group participants provided feedback about a wide range of issues related to technology—from the degree of acceptance by patients, family members, and caregivers to its benefits and challenges and the positive implications of expanding various aspects of technology into patient care activities. As indicated by the listed subthemes, this theme is characterized by a variety of categories, including desire to involve technological advances as an expected part of standard patient care.

#### Key Finding 1: Benefits of Technology

One-third of all instances of *technology-related* content were specific to the benefits of technology, that is, this subtheme tended to illustrate the extent to which various technological programs enhanced patient care or family engagement. The *benefits of technology* subtheme was primarily endorsed by corporate leaders and franchise owners and case managers, although FCGs also viewed it as a benefit. It was rarely mentioned among informal caregivers, which is more a function of the questions asked rather than not accepting or having a negative view of technology.

#### Key Finding 2: Engagement, Training, and Impact

As for the corporate leader focus group participants, they were asked to speak to making engagement between the caregiver and the person with dementia part of the care plan. The specific question was “Is [engagement] something you guys have seen in your network or something that’s on your radar as part of the training and approach, you guys are taking the dementia care?”

How do you get people to personalize care, period, and to learn and to make better days? That’s the thing. And it’s the hardest thing, but if you can figure it out with this app, we’re, we’re interested.

Corporate Staff 2, December 3, 2020

The same corporate leader reflected a similar sentiment when asked to consider their in-house training resources and their strategy for not only developing proprietary resources but also leveraging third-party resources to supplement the training:

But we have a lot of caregivers that are learners. They, just, they wanna learn more, want to know more. So that’s why we’ve offered this additional platform, that was third party. And we’ve also, we’ve put it sort of on demand, so that they can take it whenever. There, it’s convenient for them, and in any setting.

Corporate staff 2, December 3, 2020

When franchise owners were asked about an aspect of dementia care that they were most proud of, one responded:

So, I would say that’s the thing that we’re probably most proud of...is the way that we have been able to engage clients and their family members...In some cases, family members who really weren’t that involved became much more involved, um, as a result of this, of this program. So, we have family members who, you know, maybe lived at a farther distance, and this became the catalyst for them really becoming involved in the care. And just the checking in on their loved one, and it gave them a way to...create memories, by sharing old memories, and then seeing how their loved one reacts to it by the entries into the [Generation Connect] platform. It was just a really, I’m very proud of the way that this tool has helped, not only the clients, but also the family members to really create new memories and have a memorable experience.

Franchise owner and case manager, location C, November 11, 2020

#### Key Finding 3: Challenges Limit Benefits of Technology

There were also a few occasions (n=7) when various stakeholders acknowledged the benefits of technology but additionally admitted that the realization of those benefits can be somewhat undermined by particular challenges that limit their effects:

I mean, we do a lot of education on the care team and who all that involves and as family caregivers there’s the primary and the secondary caregiver and then even those, beyond that, that can support in various ways. So, I would say that it’s probably done in more like an educational forum and then I would agree, that homecare tablet really does allow for more family connection even just...the social aspects. Maybe it’s not helping with the direct care but just connecting the families. We’ve seen a lot of great success with that.

Corporate staff 1, December 3, 2020

Again, it really depends...on the caregiver or client technology. If we’re able to do a HIPAA compliant we are definitely using that, but in some cases they don’t have an iPad working, we are using the caregiver cell phone which sometimes the caregivers have technology glitches on their end and maybe you can’t get it a Zoom meeting going.

Franchise owner and case manager, location H, November 11, 2020

My clients that I worked with and did a lot of zooming with, I had to keep educating the family, like how to get things to work, right, because they got a little bit dependent on me being able to know how to do this. And they wanted to be able to use it too. And I have to keep telling them, OK, this is where you go for this. It was hard sometimes to keep saying it...There were times that my client was just got tired quickly.

FCG, location A: CG 3, November 12, 2020

However, it is reassuring that the various benefits of technology were considered prevalently among stakeholders.

### Theme 2: Care Services

#### Overview

Unsurprisingly, content related to patient care services was frequently mentioned (134 times) throughout the discussions with all stakeholder groups, as improvements in patient care underlies the objective of this technology-based project and these services. In addition to general aspects of caregiving practice, this theme encompassed attributes of clinical care, including the use of formal cognitive or mental health assessments. The care services that were mentioned also extended to family members and efforts to improve their interactions with the patient.

#### Key Finding 1: Patient and Family Engagement

Input from all stakeholder groups informed this subtheme and identified the variety of ways in which engagement with patients and families took place. Such activities range from a thorough review of the patients’ care plan with their families to helping family members interact with their loved ones with dementia, either through direct involvement or through technology:

Specifically, we walk through the whole care plan and all of the activities that we’re going to do to massage and exercise that cognition while also taking care of all the ADLs and the IADLs that we have to manage during that visit. Then we ask the family, basically, to enter into an agreement or a contract with us at that, I understand, this care plan, and I understand that this is different.

Corporate staff 1, February 2, 2021

We’ve tried to use that meet and greet kind of program that [Location G Owner 7] was talking about. But we have found that every time we set up, know, a plan or rule that says, “that’s what we’re going to do,” it doesn’t work…If we have a real serious dementia situation...that it’s going to be challenging, then we absolutely will have [Location B Dementia Specialist] go there and introduce and even stay the whole first shift with that caregiver, in order to make sure that the transition works.

Franchise owner and case manager, location B, January 22, 2021

So, she was going through a lot with her family, was going through a loss. I wanted to make sure that it was truly the family agitating her, and not just her demeanor. Over time, once I started to realize what exactly was agitating her and making her days more difficult, I initially started by bringing it up with the office...But eventually, I did start to have some discussions with [family], just to let them know that, obviously, their intentions are in the right place. But...it’s very difficult for them to see sometimes the repercussions of the things that they’re saying, the things that they’re doing, especially when their family member is in a facility or is far away because they might see them for a few minutes, but who’s there for the rest of the day. So, thankfully, I’ve, I’ve gotten a little bit better with being able to address those things with them because I have created a really good relationship with the family.

FCG, location E: CG1, November 12, 2020

I think, with my sister living in Texas, it would have been great, as [Location A: Family 1] said, to comment back, to be able to have a conversation with a caregiver. The other thing is that it would have been super helpful to know which caregiver posted what comment about my mom. Because, that would have helped us to figure out who’s using it, and who’s not.

Informal care partner, location C: family 1, November 17, 2020

#### Key Finding 2: Response to Patient

All stakeholder groups contributed information that was relevant to responses to patients, although corporate leaders mentioned this only once. This subtheme could apply to either caregivers’ and family members’ responses to patients (Table 3), with the objective of interacting with patients in a manner that does not exacerbate the patients’ emotional state or result in family members’ anxiety.

**Table 3:** Direct quotes responding to the patient from the caregiver and family member perspectives.

Staff	Caregiver response to the client	Family member response to the patient
Corporate staff	None	“So, a lot of time, and I think that’s what makes our cognitive support program special, that we really do work and educating the family. Through that process, but that also might mean that they’re not willing to accept that their loved one needs to be in this type of receiving this type of care. So, there’s a lot of just human elements that we’re dealing with in this as well.” [Corporate staff 1]
Franchise owner and case manager	“You know, it’s been nice to have access to an engagement tool where we can engage in a different way with our clients. We’ve had a couple particular situations where the caregivers have really embraced the concept of the iPad and have had one-on-one training with [Cofacilitator] from Generation Connect, which has been really awesome. And really helped them to develop and get a better understanding of how to really use the iPad to better engage the clients and just seeing the caregiver’s just come up with more creative ideas of how to use the iPad.” [Franchise owner and case manager, location B]	“We do get, sometimes, you get [a patient] who is just so resistant and the family member gives up. That mean, you know, the care recipient only wants that family member around them, uh, attempts to throw everybody out. And the family member just decides that it’s not worth, the hassle.” [Franchise owner and case manager, location B]
Formal caregiver	“So, what I try to do is, I try to redirect, I have a patient now. Who, she’ll tell me, did my husband died? And I will tell her, yes. I heard he was such a jokester, why don’t you tell me about a joke? And even though she, it’s kind of like she’s reliving the grief every time to hear that she died it’s bringing up happy memories, uh, know how he was when he was alive.” [Formal caregiver, location C: CG^a^2]	“The client’s family. Daughter in-law and son she lived with were very well educated. He was a professor. She was a registered nurse. And when I would come, they would argue with her all the time, and it made me feel like I was the bad guy because I would go along with it. If she told me, ‘the moon was made of green cheese,’ but they would argue all the time with her. And I know, [my manager], she gave them lots of videos and lots of links to look at. but they just wanted nothing to do with that. If they were there the whole time that I was there with them, I was exhausted mentally and physically, and I mean there were times I left there crying, because I was so upset with them. And then, the client that I had just before this one, I couldn’t have had a better support system with a son and daughter-in-law, I mean, it’s just phenomenal. She was a teacher and I forget what he did, architect. But, you know, I just, yeah, I just feel the mind has to be open to the heart or vice versa to be able to understand the daughter of my client now.” [Formal caregiver, location A: CG2]
Informal care partner	“Building on what [Location F: Family 1] said when I first noticed changes in my dad. I wanted him understand things logically. So, I would argue with him and try to make him see my point of view, which only led to not good situations. And my case worker through [home care company] sort of suggested to redirect. If we’re talking about something, mentioning something else, can redirect the conversation’s show that his mind would go on something else, not what we were talking about, and gradually, I learned to just go with the flow.” [Informal care partner, location E: Family 1]	“I did a little bit of reading, but to me, it just started feeling that [it] was the only choice, you know, because I really wasn’t interested in having an argument with my dad, you know. I didn’t need to win him over to my point of view. So, I think that was just a little bit, for me, it just seemed like a natural thing to do. I think my sister, I think she, just, over time, also saw that there wasn’t any percentage in it...we were not able to convince him of this factual thing, or that actual thing, and so I think she’s, that, just over time, she also kind of, I don’t know, maybe she saw me do it, but it also could just have come to her. She just has a different personality. She’s a more, she’s a more logical, analytical person so that you know that was her, her go-to’s – we’ll lay out the facts. I have a different way of being in the world. And so, I’m more interested, I’m more looking like, ‘well, what’s, you know, what’s the relationship here, what’s connected?’” [Informal care partner, location F: Family 1]

^a^CG: caregiver.

### Theme 3: Data, Documentation, and Outcomes

#### Overview

Corporate leaders and franchise owners and case managers were much more likely to offer information about *data, documentation, and outcomes* than FCGs and informal care providers. In total, all stakeholder groups referenced this theme 89 times. Overall, such feedback related largely to the use and value of cataloging changes in patients’ mood or behavior to evidence the effects of treatment. However, there was also an interest in communicating about sharing information and resources and in factors that can undermine such sharing to caregivers or family members.

#### Key Finding 1: Need to Document Outcomes

This subtheme reflected the same pattern of responses as the overall thematic category, with only the corporate leader and franchise owner and case manager stakeholder groups having input about the *need to document outcomes*. Much of this feedback was elicited from direct questions about efforts to quantify patient care outcomes and the limitations of such efforts:

Our operating system is the one that every home care company uses, and so we track a care plan and we track the tasks that are done every day. But where the outcome is...the seniors’ needs, or goal, and the family’s goal. And that’s an, a text box. And so, that’s one issue where we’re, you know, we can’t really match what the goal is with the “what’s happening.”...also the way our systems are set up – they’re not built, for pulling outcome data, [and] it’s hard to track hospitalizations when there’s nowhere to capture where, when they had their last hospitalization before they became one of our clients, for example.

Corporate staff 2, December 3, 2020

We track incidents and falls for the Department of Health. We have to have an incident book if somebody falls or something happens. So, we try to track those to see, you know, just for that reason, clearly, if somebody is falling a lot, we try to figure out why and make changes on it, you know, so, we’ll just keep track of it in our system, client file. No, that’s about it.

Franchise owner and case manager, location G, November 11, 2020

#### Key Finding 2: Feasibility of Using Technology to Document Outcomes

Although there was prevalent recognition of the utility of outcome measurement, 1 focus group member brought up a specific constraint relating to this objective:

But, right now, so we actually input into our operating software, ways that they could track...mile markers...But our Franchisees actually have to go in and check the mile markers. And then they have to do the assessment repeatedly, so that they can see where they’re at on the mile markers. And we actually have care plan associated with all four of those different ability levels that a client will go into, based on where they’re at in the journey, based on their ability level, and what they’re likely able to do, and what they’re likely not able to do, based on their disease progression. The problem is tracking. So, right now, to [corporate staff 4]’s point, I can’t get that information to a family unless our franchisees regularly monitor assess and are input that information into our software.

Corporate staff 1, February 2, 21

In addition, when questioned about the potential for standardization of assessment forms in the system, as a means to consistently compare among franchises, it was admitted by one stakeholder that standardization was not feasible at the present time: *“*But there is no standard assessment form either” [Corporate staff 2, December 3, 2020].

### Theme 4: Cost, Finance, and Resources

*Cost, finance, and resources* represents the most unique theme (with 82 identified instances), where most of the relevant content was obtained from corporate leaders and franchise owners and case managers. In addition to the issues most relevant to this theme, other related content involved discussions about the benefit of having staff members who are dedicated to specific activities or responsibilities, factors influencing staff turnover, and MA and other reimbursement programs, most of which involve some amount of administrative burden.

This most frequent subtheme was derived from focus group questions related directly to the topic of MA. Such questions elicited quite elaborate responses related to the pros and cons of this reimbursement policy, which is illustrated most thoroughly with the following corporate leader stakeholder feedback:

I mean, the biggest issue right now is just reimbursement, and hours, right? So, you’re exactly right, Your last comment. It is true. There’s, we’re seeing more demand than we’ve ever seen. Why in the world would we waste the time on Medicare Advantage when the reimbursement isn’t? We can’t pay our caregivers, so there’s no margin. You’re getting 20 hours, a quarter of care, which really doesn’t have any sort of impact on outcome anyway. The only reason that we’re even, and we do participate in a few Medicare Advantage plans, but ultimately, the only reason is to collect the data on why shouldn’t we do it anymore.

Corporate staff 3, February 2, 2021

We, actually, I should say we billed, but did not get paid through some Medicare Advantage plans. And the motivation is, it’s really, it’s kinda like long term care in my mind. You’re billing a policy that somebody has paid into and it helps them get the care they need, right? That type of thing. We had, previously, probably, most of the people on here don’t do this anymore. We had done an assignment of benefits or policies and we’re phasing that out. Some of them, you know, obviously will keep the ones that we still do an assignment of benefits for, but we won’t be doing that anymore, because an insurance company will always pay their member before they’ll pay us. So, that’s one of the reasons we’re phasing it out. But I think the idea behind it is it gives care to people that might otherwise not be able to afford it or might not even want - if they understand the benefit. It might help them remain independent and at home longer, if they utilize those benefits that they have.

Franchise owner and case manager, location F, January 22, 2021

### Theme 5: Resources for Caregivers

Unsurprisingly, content related to patient care services was frequently mentioned during the stakeholder discussions (70 times), as improvement in patient care is the objective of this technology project and these services. Despite the different subthemes, the concepts within this theme universally highlighted the importance of training and support as a key function in maintaining the ongoing use of the Generation Connect platform.

Despite the general view that technology has its uses and advantages, it was evident from focus group responses that home care provider staff and family members have a *need for training or education*, especially in relation to technology. Although there were particular questions designed to elicit information about education or training needs, some of the content was forthcoming naturally when describing other aspects of patient care. The need for training and education was endorsed relatively evenly across corporate leaders, franchise owners and case managers, and FCGs; however, informal caregivers also provided relevant feedback about their need for dementia-related knowledge. Table 4 details the perspective of the focus group participants related to training and education related to technology and caregivers or family members’ knowledge about dementia.

**Table 4:** Direct quotes responding to the need for training and education related to technology or knowledge about dementia.

Staff	Training or education related to technology	Training or education related to caregivers’ or family members’ knowledge about dementia
Corporate staff	“But I do know that that the caregivers play a big role and there’s a lot of training that the office does for the caregivers to say, you know, this is what the tablet is. This is how you can engage. And the caregivers, I think there’s an app specifically for the caregiver on the tablet, so they’re interacting with it regularly for like things like clocking in and out, and those types of things, so that automatically I would imagine a lot of times prompts engagement too with the client, because the caregiver’s interacting with it so regularly. I think another neat function is that the tablet has like scrolling pictures on it, so when it’s charging even, it’s kind of like a rotating picture frame. So, I think that that in and of itself to kind of prompts interaction with it, especially if there’s a new photo that they haven’t seen before. But I would imagine for those living with dementia, probably is more caregiver interaction.” [Corporate staff 1]	“I would say that another addition, though, to the proprietary training following the Alzheimer’s Dementia Care evidence-based practices was about assessments, which we always do, but this training that we’ve updated, took that to another level and sort of explained how important it is. That’s what we’ve always explained, that it is important. But now, we’ve actually said, here’s how we can do it.” [Corporate staff 2]
Franchise owner and case manager	“I think the biggest thing for us is just continuing to keep the education piece on all staff. So, our frontline workers being trained in dementia care, because you never know when you’re going to have an opportunity, where current client, they’re taking care of, starts experiencing, signs, and symptoms. So, you know, I think, the, earlier we catch it on, then, the better partnership we have with the frontline staff and the families. And that’s kind of a goal that I’ve been doing in the community, you know, barring this year, is educating people and, you know, trying to bring technology into it.” [Franchise owner and case manager, location H]	“Now one of the things we’re very proud of is the fact that we do a lot of one-on-one dementia care. I’m a certified dementia trainer with the Alzheimer’s Association, so all of our caregivers go through training with me before they can put out in the field. We also have great mentoring programs.” [Franchise owner and case manager, location D]
Formal caregiver	“So, we’ve just had a lot of success with it. The only difficult part has been making it work with providing links for the family. I, I don’t know if maybe there’s some type of re-education that can go on with the family, and using the care team Connect app. Or if there’s just...because I, I navigate the relatively well. So I don’t typically look into it. I don’t know if you guys have like a tutorial section of the apps, that the family would be able to use to educate themselves more on how to navigate the app. But I know that sometimes, they’ve had difficulties, so, they’ve had a hard time finding where to upload photos, where to click the link, so that they can join Zoom calls, But, but, overall, I mean, the Zoom calls and face timing has gone really, really well.” [Formal caregiver, location E: CG^a^1]	“So, thankfully, I’ve gotten a little bit better with being able to address those things with them because I have created a really good relationship with the family. It kind of has fallen back on me. It was just having those conversations with them. Sometimes it works, sometimes it doesn’t. It’s kinda one of those hit and miss things, but a lot of it falls back, just to the fact that the families usually just don’t have the amount of education that we do, and aren’t always open to the education that we can give them, just because they don’t, they don’t really see the, the depth, that it really has. They don’t see how much we’ve actually had to learn about it. They only see a surface level of us telling them, that, their actions are making the family upset. Your words are making them upset, then they get offended. So, I feel like, I feel like if I had more resources to be able to give them more resources to be able to direct them to, it would make things a lot easier. I just said that resources were really important for them.” [Formal caregiver, location E: CG1]
Informal care partner	“Would you be able to load videos like a video of my grandson running through the yard because I have not been able to figure out how to post a video? I can do photos, but I have not figured out videos...I just need a lesson.” [Informal care partner, location A: family 1]	“So, I do think that there’s so many things about my parents getting older and having health problems, and these mental problems, and all of these things. And I, often, my sister today, we’ll just look at each other, will say, we cannot be the only ones, we’re not the only ones. And yet, it’s so hard, it’s so hard to find. Where is that training? Where are those resources? It is, it’s not, it’s not at all easy, and, as this disease becomes, I think, at some point, it will be epidemic. We’re going to live, everyone is going to live so long that our population is going to be filled with people who have this disease. And maybe just the sheer volume of folks will help bring. Bring this topic and, and these, this, this training, and these ideas more into the mainstream, I have to say, I, I was very uncomfortable telling anyone that My dad has Alzheimer’s, at all for...I felt a lot of shame about that, which is really unfortunate.” [Informal care partner, location F: family 1]

^a^CG: caregiver.

### Other Relevant Findings

A number of other issues warranted discussion because they were identified frequently from a particular stakeholder group or else represent unique or noteworthy implications. Multimedia Appendix 1 summarizes these issues by stakeholder group and provides exemplary quotes.

## Discussion

### Principal Findings

The focus group findings highlight the complex and dynamic implications of providing care for persons living with dementia in their homes. More importantly, this study contributes to the growing literature on how technology can support care for people with dementia living in the community [18,19] and the importance of a user-centered design approach [20]. Overall, the stakeholder group participants share a widespread belief about the benefits of technology in general and about the Generation Connect platform, specifically with regard to the platform’s support efforts for caring for individuals with dementia in their homes. In general, this finding supports the benefit of technology acceptance for promoting technology use [21-24]. It is clear that in this case, familiarity breeds acceptance, with many FCGs reporting using the Generation Connect platform during their shifts. In doing so, they tended to notice a range of improvements in client behaviors and client relationships. Our results reinforce earlier findings suggesting that direct interaction with technology is associated with increased familiarity [25] and that increased engagement is related to improved outcomes [26]. Most importantly, FCGs believed that iPad use helped them connect with clients and facilitated enjoyable experiences. These results were found although the Generation Connect platform was largely used with clients in later stages of dementia and with more severe symptoms.

Corporate members and franchise owners were particularly enthusiastic about the possibility of providing individualized care, and the platform under development reinforces this approach with its personalized content and ability to expand patient personalization options. Such results support the key value of personalization when designing technology for people with dementia [27], which is associated with improved social engagement, mood, activities of daily living, and the caregiver and participant relationship [14,28,29]. More generally, the direct involvement of key stakeholders aligns with recommendations from an earlier position paper calling for the direct involvement of stakeholders when designing technology for people with dementia to support meaningful use [18]. However, formal and informal caregivers have somewhat different opinions about the benefits of individualized care on patient outcomes in the Generation Connect platform. FCGs considered most of the app features to have potentially positive implications. However, the ability to routinely share updates with family was not believed to be as important as other platform characteristics. This finding may represent an area for further consideration or exploration to understand why this feature of the Generation Connect platform was not as widely accepted. Informal caregivers’ opinions were similar, in general, about the benefits of the Generation Connect platform regardless of how often they used it. Understanding the advantages of individualized care seems an essential issue for convincing FCGs to adopt it into their routine care practice.

Despite the general acceptance of the Generation Connect platform, challenges need to be considered and addressed for broad and successful adoption. For example, a noticeable perception from informal caregivers was that less frequent users tended not to see a difference in caregiver interactions. This finding suggests the need for more direct communication with informal caregivers and even FCGs about the potential immediate and sustained benefits of the Generation Connect platform that can be derived from repeated use of the technology. In addition, acceptance of technology was not universal. Oftentimes, patients’ use of the Generation Connect platform was determined by their previous comfort with technology, but family members certainly indicated that they could play an important role in assisting patients so they could benefit from engaging with content on the device.

Feedback about challenges and limitations to the benefits of technology largely reinforced the need to provide informational resources to mitigate the influence of knowledge deficits. Across the stakeholder groups, participants generally understood that education or training is necessary to better prepare caregivers for the broader use of technology and that it would be beneficial for some family members to receive similar content. In particular, corporate staff and franchise owners consistently expressed a belief in the importance of training to ensure that FCGs can provide appropriate and clinically valuable care to individuals with dementia, which could include patient engagement through technology. As a result, strategic training efforts are required to incorporate the Generation Connect platform into standard care practices.

Although it is feasible to address many of the identified technology-related challenges and shortcomings through additional education, cost remains a relevant limiting factor. Identified cost issues ranged from insufficient reimbursement for care services to the financing of technology hardware and internet services. However, external environmental changes targeting expanded broadband access, or increasing technology affordability and accessibility, may reduce cost burdens.

Finally, corporate members and franchise owners considered patient outcomes and the documentation of such outcomes as worthy of investigation, which can be aided by the Generation Connect platform. However, feedback suggests that corporate members may require more direct evidence of the benefits of using the Generation Connect platform to record and track client outcomes before committing to the technology. Franchise owners, on the other hand, not only recognized the importance of tracking client outcomes but also were more likely to already be engaged in such activities.

### Strengths and Limitations

The focus groups conducted for this study phase provided important insights into the mindsets of various key stakeholders about the viability of using tablet technology and the Generation Connect platform to facilitate approaches to reduce ADRD symptoms and strengthen caregiver and persons living with dementia engagement. However, the number of focus groups was limited, and many of the focus group participants had prior experience using the Generation Connect platform. This purposeful participant selection was considered necessary, given the objective of this project phase to elicit feedback about the Generation Connect platform that would help guide its construction content refinement and expanded adoption.

In addition, response bias is possible because of the stakeholder focus groups. However, Generation Connect staff attempted to mitigate potential bias by (1) using a standardized interview protocol emphasizing that the forums were meant to elicit feedback that should relate both to positive and negative experiences and (2) structuring focus groups to control for the effects of organizational hierarchy (eg, franchise owners were provided separate forums from FCGs who may work within those franchises). Finally, more comprehensive feedback from the FCG focus group could have been attained through an intentional recruitment strategy that included FCGs who declined to use the Generation Connect platform when presented the opportunity, to compare to the feedback from FCGs with experience using the platform. Similarly, a few home care franchises failed to operationalize the Generation Connect platform in the pilot testing session. Including these individuals in the focus group could have resulted in more comprehensive feedback and insights into the limitations on or barriers to the adoption of the platform at the franchise level.

### Conclusions

It is encouraging that the Generation Connect platform can help address many of the issues identified through stakeholder focus group interviews. Further development of the Generation Connect platform capabilities for nonclinical home care will be informed by stakeholder feedback to reduce the burden of caregiving for persons living with dementia, evaluate changes in cognition, preserve functional independence, and promote engagement between persons living with dementia and caregivers. Successful completion of this overall project, leading to a finalized platform, is planned as the basis for a larger NIH SBIR phase 2 clinical research trial to assess the efficacy of evidence-based interventions and the market viability of the Generation Connect platform. Specifically, the principal aim of the phase 2 project will be to quantify the economic impact (eg, reduced hospital or emergency department admissions, falls, and care transitions) and clinical outcomes (eg, decreased anxiety, depression, and isolation) and support or accelerate home care network efforts to standardize data collection around these key outcomes. Demonstrated effectiveness through a clinical trial would reinforce the Generation Connect’s go-to-market objective of commercializing the Generation Connect platform in the home care industry as a viable ICT solution to improve clinical outcomes, reduce turnover, extend client length of stay, and support emerging MA plans.

This study revealed key discoveries that are essential to consider for future projects, which are as follows: (1) early intervention and collaboration between FCGs and family members who are providing care; (2) great variability exists across national franchise networks in their ability to adopt and commercialize technology solutions; and (3) providers have systems in place to track clinical data and outcomes, but they are limited in scope and lack standardization and interconnectedness. Importantly, the insights gained from this study will prove critical to informing our approach for the phase 2 outcomes research and future commercialization efforts.

## Appendix

Multimedia Appendix 1 Exemplary quotes associated with other issues identified from the focus groups.

## Abbreviations

ADRD: Alzheimer disease and related dementias
FCG: formal caregiver
HIPAA: Health Insurance Portability and Accountability Act
ICT: information communication technology
MA: Medicare Advantage
NIH SBIR: National Institutes of Health Small Business Innovation Research
TECH: Technology-Enabled Caregiving in the Home
