# Supplementary material for: Refining a Digital Therapeutic Platform for Home Care Agencies in Dementia Care to Elicit Stakeholder Feedback: Focus Group Study With Stakeholders
Source: JMIR Aging. 2022 Mar 2;5(1):e32516. doi: 10.2196/32516 (PMC8928048; doi:10.2196/32516)
Supplement: Multimedia Appendix 1 [file aging_v5i1e32516_app1.doc]

**Multimedia Appendix 1**. Exemplary Quotes Associated with Other Issues Identified from the Focus Groups

| Additional Identified Issues | Stakeholder Group^1^ | | | Exemplarity Quotes |
| --- | --- | --- | --- | --- |
|  | FC | FO/CM | CL |  |
| Use and Value of Personalized Content | X | X |  | I think the most thing with care team connect, has been a really valuable thing to offer to our clients simply because with dementia, like what you mentioned with the music is something that our clients really benefit from as well as the games have been popular and a lot of the family's involvement with uploading pictures that they can reminisce together so. Adding that to our Personalized care plan for dementia has definitely been a huge benefit, so we're very thankful for the opportunity to be a part of this program. (Franchise Owner/Case Manager, 12/3/20)  My client, like she was a fantastic piano player. And I find that when she gets a really agitated. I get the iPad and do piano music to calm her down and she really enjoys that. So, in that aspect, the iPad's been wonderful. (Formal Caregiver, Location B: CG3) |
| Personal Challenges of Technology | X | X |  | Our seniors can’t initiate, they don't remember to, and they don't know it's not part of their routine. So, something has to do that for them. It's either a human being or some piece of technology that we're able to initiate or just initiates based on a time schedule on every device has, you know, the right time. So, it happens. Now, what you don't want to do, a guess freak people out, but, you know, it's still a basis by which, you know, it's not them that start that process, I don't know. It's hard. It's a hard one because every situation is different, right. (Franchise Owner/Case Manager, Location G)  Are able to upload videos onto the app?  Is the family able to upload videos onto the app, or is that not a feature that's available, because I've tried and haven't had any success with it, but I could just be utilizing it wrong? [Facilitator question: Do you mean the videos that you've taken, or videos that you copied? And wanted to put in the app from YouTube?] No videos like that, I've taken, because my clients wanted to send messages to her family before, like birthday messages, and I just haven't been able to upload personal videos from hers.  And I also know that her family has wanted to upload some videos of some of her great grandkids and just old home videos that they haven't been able to upload. (Formal Caregiver, Location E: CG1) |
| Implementation of Individualized Care |  | X |  | Well, I mean, just the very basics individualized care plan Is one page and our dementia care plan is three. I know that may seem like not the information you're looking for, but there should be much more higher-level detail for our dementia. Think about their background. Where were they born? What is their marital history? What is their military history? What were their hobbies? What was their religious affiliation? Are their behaviors or issues that they struggle with? How do they communicate? How they make themselves understood? What time did they go to bed? What time do they get up, do they struggle at night? What kind of assistance do they need physically in all of their ADL? We go through all of those at a much higher level of detail for our dementia. (Franchise Owner/Case Manager, Location C) |
| Demonstration of Value |  |  | X | There should be a point where, if we assess a client, we know where they are on the cognitive spectrum, and then we could be able to determine, OK, [Facilitator 03] we're signing up your mom today. This is where she is. Her progress in the next years will Get Her down the road, too, whatever the mile marker is. But, having a caregiver here, well, hopefully, slow, if the trip will take longer with us versus without us. And so, we can see exactly. What our: Yeah, here we are today, and this is where she would be [Corporate]. This is where she is with [Corporate]., as she's closer to the mile marker where we started, versus down the road progressively, and then that needs to be, that needs to be placed into some element of A patient summary in the sense because most of the seniors don't live with their, with their sons and with the daughter. (Corporate Staff 4) |
| Need and Value of Sharing Information/Resources |  | X |  | What we usually do is, we have an incident report that frontline staff would fill out. So, if there was a particular incident where the client hit the caregiver, or no lock themselves in the afternoon, or whatever it might be, every story out. Second prize, all the time, a new one, then what that goes directly to the case manager, in that case manager can irradiate that paperwork. Obviously, most of a caregiver call in advance, so we can inform any other caregiver in going that way. They're on alert. And then, they finally come to me, to look at different trends. You guys were talking a little bit before, like, other trends like a lot of falls going on, how could we look at that as a team to break that down? Should a physical therapist be called in? So, we're always looking at ways to improve the outcome, and prevent any more incidents. So, that's kind of how our office handles that. We are in a licensed state, so are only required to report really unusual incidents. Usually, things that end up with a medical emergency, or history of abuse, or things like that. (Franchise Owner/Case Manager, Location H)  The care plan is, we, utilize Clear Care and we utilize a Clear Care Go Apps and a care plan itself actually is on the caregiver's smart phones. Then we also leave a hard copy of the care plan in the home. And our communication book, but the nurse will review the care plan with the family member. And we are required to have the family members sign off on the care plan. But, as far as other internal documents, as far as keeping track of our falls and hospital re-admissions, that's not open too, you know, to caregivers, to be able to readily access in Clear Care. Because that's really where we access all the data from. (Franchise Owner/Case Manager, Location E) |
| Cost and Staff Turnover Issues or Cost Options^2^ |  | X | X | Ours usually have to do with finances at the time, because, obviously, we're legitimate spend down for Medicaid, but if they're going to eventually end up in a facility, they need to have that 2 or 3 years, or sometimes four of a Medicaid spend down of private pay. So if you get into a point where they do need a live-in or they're awake 24/7, and they need 24/7 care, that's very expensive. So, families look at it that way. Usually, it isn't because the family or the client isn't getting what they need from the service. It's more monetary. That they're making those decisions. (Franchise Owner/Case Manager, Location F)  I believe it's the number of hours, if you have somebody and you're willing to let them work 10, 20 overtime hours, they'll stay, I think, we offer, I think, a very competitive rate. We're above minimum wage, certainly pay $15 an hour, but I think it's they're always looking for a better deal so they can get a better deal somewhere else, a private client, they will take it. But if we keep them working with enough hours, they'll stay with us. (Franchise Owner/Case Manager, Location G)  So, I think if you look, what I thought was most successful is, obviously, in order for your customer base, for an owner to successfully implement certain initiatives, if the financial burden is not there and evenly distributed across the entire network of check of clients, then it takes the financial risk off. Right? So even if it's 40 bucks a month for a unit, by the time you break it down, you get 40 clients, you get 40 units and you do the math. For the most part, that might be an increase of 25 to 45 cents per hour. Well, at the end of the day, that's nothing. But if it, if it's spread across everyone equally, and if you have a very strong private pay population of business, you can do so. You can raise your rates when you raise them, and you can state that this is a part of, this is how we bring care to your home. If it's with the unit. If it's with a tablet, as [Corporate Staff 1] was talking about, with telemedicine, we put this in everyone's home. You will get a call once a day, twice a week, three times a week, for us touching base, and this is how we provide to you. People, they come to us as the expert. We shouldn't be asking them, what do you want? And so many times you, well, what type of services does Mom need? Well darn it. If I knew that, I wouldn't be asking you for help. Right? I mean, that's just we just have to be in a more authoritative position. (Corporate Staff 4) |
| Administrative Burden |  | X | X | So many hours of care, and this, these types of services equal as much of the dollars, so on and so forth, I just felt like right now, Um, it seems like a pretty big, like owners are saying kind of a hassle. I kinda. it's too much admin work, it's too hard to do all that right now. (Corporate Staff 2)  So, we work with a few different sources, we work with the veterans through, either through the VA Hospital itself, or through the Veterans Care co-ordination companies, and we also in a very, very small percentage - we do still take the Medicaid waiver, in a very minimal amount, because for the same reason, that's where you have no desire to take Medicare Advantage reimbursement, as well The amount of time it takes to get paid is ridiculous. And now that they switched to MCOs, it's made it even more impossible. (Franchise Owner/Case Manager, Location D) |

1. FC = Formal Caregivers; FO/CM = Franchise Owners/Case Managers; and CL = Corporate Leaders
2. Cost Options was an issue for corporate leaders while franchise owners and case managers were concerned with cost and staff turnover
